# Fitting the pieces of the puzzle together: a case report of the Dunnigan-type of familial partial lipodystrophy in the adolescent girl

**DOI:** 10.1186/s12887-016-0581-2

**Published:** 2016-03-14

**Authors:** Paulina Krawiec, Beata Mełges, Elżbieta Pac-Kożuchowska, Agnieszka Mroczkowska-Juchkiewicz, Kamila Czerska

**Affiliations:** Department of Paediatrics, Medical University of Lublin, Racławickie 1, 20-059 Lublin, Poland; MEDGEN Medical Center, Orzycka 27, 02-695 Warsaw, Poland

**Keywords:** Familial partial lipodystrophy, *LMNA* gene, steatohepatittis

## Abstract

**Background:**

Familial partial lipodystrophy of the Dunnigan type (FPLD 2) is a rare autosomal dominant disorder caused by the mutations of the lamin A/C gene leading to the defective adipogenesis, premature death of adipocytes and lipotoxicity. FPLD 2 is characterized by a progressive loss of subcutaneous adipose tissue in the limbs and trunk, and accumulation of body fat in the face and neck with accompanying severe metabolic derangements including insulin resistance, glucose intolerance, diabetes, dyslipidemia, steatohepatitis. Clinical presentation of FPLD 2 can often lead to misdiagnosis with metabolic syndrome, type 2 diabetes or Cushing syndrome.

**Case presentation:**

We report a case of a 14-year-old girl admitted to the Department of Paediatrics due to chronic hypertransaminasemia. On physical examination the girl appeared to have athletic posture. She demonstrated the absence of subcutaneous adipose tissue in the extremities, sparing the face, neck and gluteal area, pseudo-hypertrophy of calves, prominent peripheral veins of limbs, massive acanthosis nigricans around the neck, in axillary and inguinal regions and natural skin folds, hepatosplenomegaly. Laboratory results revealed hypertransaminasemia, elevated γ-glutamyltranspeptydase, and dyslipidemia, hyperinsulinaemia with insulin resistance, impaired glucose tolerance, and hyperuricemia. Diffuse steatoheptitis in the liver biopsy was stated. Clinical suspicion of FPLD 2 was confirmed genetically. The pathogenic mutation, R482W (p.Arg482Trp), responsible for the FPLD 2 phenotype was identified in one allele of the *LMNA* gene.

**Conclusions:**

Presented case highlights the importance of the holistic approach to a patient and the need of accomplished collaboration between paediatricians and geneticists. FPLD 2 should be considered in the differential diagnosis of diabetes, dyslipidemia, steatohepatitis, acanthosis nigricans and polycystic ovary syndrome.

**Electronic supplementary material:**

The online version of this article (doi:10.1186/s12887-016-0581-2) contains supplementary material, which is available to authorized users.

## Background

Lipodystrophy refers to a wide array of congenital or acquired syndromes manifesting with the general or partial absence of subcutaneous adipose tissue, which are frequently associated with metabolic derangements [1]. Familial partial lipodystrophy of the Dunnigan type (FPLD 2; OMIM #151660) is a rare autosomal dominant disorder defined by a progressive loss of body fat in the limbs and trunk with an accompanying accumulation of subcutaneous adipose tissue in the face and neck leading to severe metabolic consequences i.e. insulin resistance, glucose intolerance, diabetes, hyperlipidemia, steatohepatitis [1]. Patients with FPLD 2 may be misdiagnosed with metabolic syndrome, type 2 diabetes or Cushing syndrome [1].

FPLD 2 is caused by the mutations of the lamin A/C gene (*LMNA*) located on chromosome 1q21-22 [1, 2]. The *LMNA* gene encodes A-type lamins - proteins, which contribute in the maintenance of nuclear structure, transcriptional regulation and heterochromatin organization [3]. The majority of LMNA mutations are heterozygous, missense mutations of 482nd codon (with variable aminoacid substitution; p.R482W/Q/L) leading to the defective adipogenesis, premature death of adipocytes and lipotoxicity [4, 5].

We present a unique case of an adolescent girl who remained under the comprehensive supervision of dermatologist due to acanthosis nigricans and gynaecologist due to suspicion of polycystic ovary syndrome. She was admitted to the Department of Paediatrics with chronic hypertransaminasemia at the age of 14 years old. The liver biopsy showed features of steatohepatitis. However, it was not the final diagnosis but just another piece of the puzzle. Medical history, clinical phenotype and the results of additional tests strongly suggested FPLD2, which was confirmed by molecular testing. Although our patient remained under the comprehensive supervision of paediatrician, dermatologist and gynaecologist, the final diagnosis was stated at the age of 14 years. It should be stressed, that despite young age of our patient, the delay in FPLD2 diagnosis led to severe metabolic derangements and decreased quality of life. We present that case to highlight the importance of clinical acumen and holistic approach to a patient based on thorough medical history and careful physical examination. We would like to emphasise that the recognition of steatohepatitis should alert one to the possible diagnosis of rare metabolic disorder including FPLD2. We believe that present case report will improve the awareness of FPLD2 among paediatricians and result in earlier diagnosis of that disorder.

## Case presentation

A 14-year-old Caucasian girl was admitted to the Department of Paediatrics, Medical University of Lublin, Poland, for hypertransaminasemia of six months’ duration, which was stated for the first time in laboratory tests performed due to acanthosis nigricans by dermatologist.

**Fig. 1:**
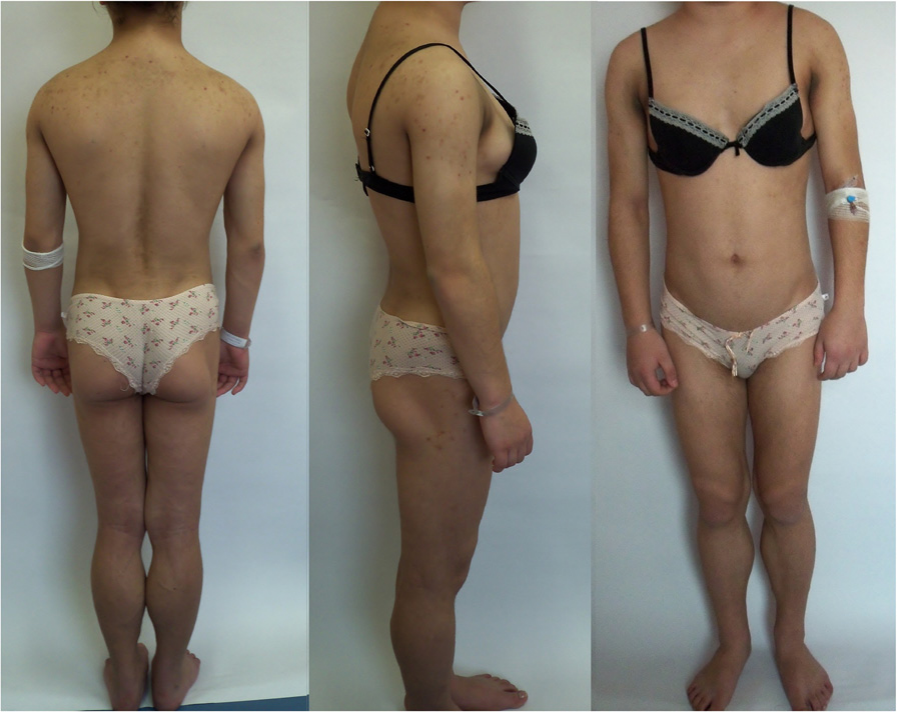
Patient with the Dunnigan-type familial partial lipodystrophy

The girl was born preterm at 36 weeks of gestation by caesarean section after uncomplicated first pregnancy. Her birth weight was 2,050 g. At the first minute after birth the Apgar score was 9. The neonatal period was complicated by prematurity problems i.e. pneumonia, sepsis, anaemia and prolonged jaundice. Afterwards, normal mental and physical development was observed. Till puberty she had no relevant medical history. Since menarche which occurred at the age of 11 years, she noticed gradual loss of body fat and acanthosis nigricans around her neck, in axillary and inguinal regions. She subsequently developed an athletic appearance (Fig. 1). She also suffered from oligomenorrhea. Changes in the physical appearance did not disturb her, because her mother and grandmother presented similar silhouette. However, both mother and grandmother of our patient deny any medical conditions and they did not consent on any further diagnostic evaluation.

**Fig. 2:**
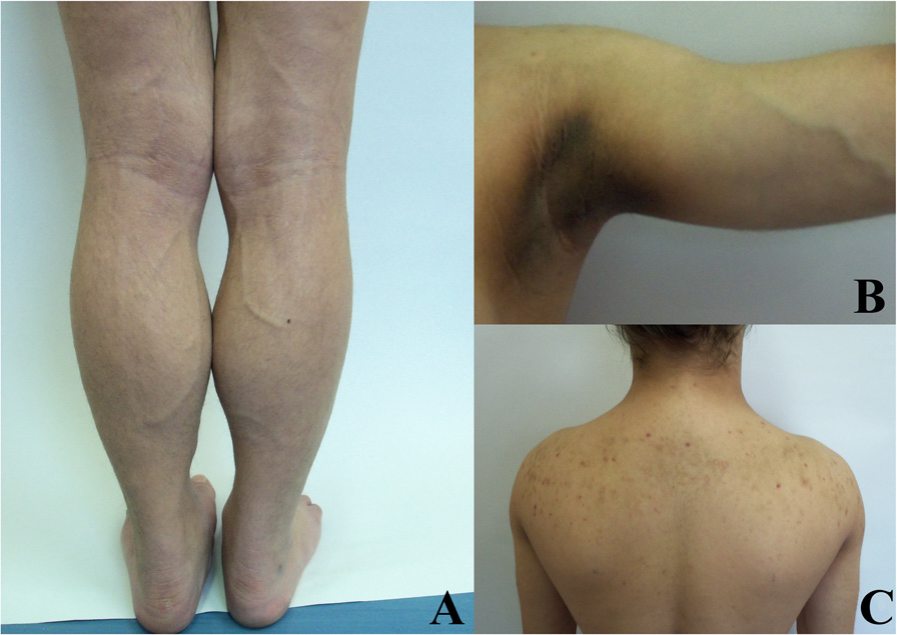
Selected features of the Dunnigan-type familial partial lipodystrophy. **a** Pseudohypertrophia of calves, prominent peripheral veins of lower limbs. **b** Massive acanthosis nigricans. **c** Acanthosis nigricans, acne lesions on the trunk

On physical examination, the girl appeared to have athletic posture. She demonstrated the absence of subcutaneous adipose tissue in the arms and legs, sparing the face, neck and gluteal area, fat accumulation in the pubic and vulva area, hypertrophy of skeletal muscles particularly of calves, prominent peripheral veins of the limbs, hirsutism, hypomastia. Massive acanthosis nigricans was seen around the neck, in axillary and inguinal regions and in natural skin folds. Acne lesions occurred on the face and upper trunk. On both shins small subcutaneous lipomas were palpable. The liver edge was palpable about 1.5 cm below the right costal margin. Spleen was no palpable. She had elevated blood pressure (155/80 mmHg). Selected features of FPLD 2 in our patient are presented in Figure 2.

Her weight was 60.3 kg (75-90^th^ percentile), height 162 cm (50^th^ percentile), body mass index 22.5 kg/m^2^ (75-90^th^ percentile) and waist to hip ratio was 0.88. She had disproportionately short limbs compared to the trunk, with height to leg length ratio almost 2:1. Bi-acromial distance (35.4 cm) was greater than bi-trochanteric (26.7 cm), giving her impression of large build. The sum of three skin folds was at 25^th^ percentile. The chest circumference was excessive (>97^th^ percentile), with large chest width and depth (90-97^th^ percentile). In the bioelectrical impedance analysis (BIA) total fat mass was 21 %.

Laboratory results revealed hypertransaminasemia, elevated γ-glutamyltranspeptydase, hypercholesterolemia, hypertriglyceridemia, hyperinsulinaemia, insulin resistance, impaired glucose tolerance, decreased adiponectin, increased creatine kinase MB and hyperuricemia. Detailed diagnostics was performed to determine the cause of hypertransaminasemia. We excluded viral hepatitis caused by hepatitis B virus, hepatitis C virus, cytomegalovirus, Ebstein – Barr virus and human immunodeficiency virus, autoimmune hepatitis, α1-antitrypsin deficiency, and Wilson’s disease. Table 1 presents selected laboratory results of our patient.

**Table 1:** Selected laboratory results of the patient

Parameter	Result	Reference range
Bilirubin [mg/dL]	1.09	<1,5
ALT [U/L]	222	<23
AST [U/L]	97	<25
GGT [U/L]	120	<23
Creatinin [mg/dL]	0.6	0.5 – 1.1
Urea [mg/dL]	29	19 – 49
Uric acid [mg/dL]	8.6	5.7
Oral glucose tolerance test		
Fasting plasma glucose [mg/dL]	80	≤126
30 min glucose [mg/dL]	119	
2 h glucose [mg/dL]	154	<140
Insulin after glucose load		
Fasting plasma insulin [mU/L]	77.1	3-25
30 min insulin [mU/L]	375.6	3-25
2 h insulin [mU/L]	920.9	3-25
Insulin:glucose ratio	0.96	
Homa-IR	15.23	
Quicki	0.17	
HbA1C [%]	4.6 %	4-6 %
Fructosamine [μmol/L]	265	100-285
C-peptide [ng/mL]	7.77	0.81-3.85
Lipids profile		
Total cholesterol [mg/dL]	230	115 – 190
HDL [mg/dL]	43.7	>40
LDL [mg/dL]	132	
Triglycerides [mg/dL]	271	<150
Adiponectin [μg/mL]	2.2	>10
Leptin [μg/L]	7.7	2.43-28
Anti-Hbe antibodies	negative	negative
HBs antigen	negative	negative
Anti-HCV antibodies	negative	negative
Anti-EBV antibodies	negative	negative
Anti-CMV antibodies	negative	negative
Anti-HIV antibodies	negative	negative
α-1-antitrypsin [g/L]	1.3	0.9-2.0
Ceruloplasmin [g/L]	0.2	0.16-0.45
Serum copper [μg/L]	1018	800-1550
Autoantibodies		
ANA	1:40	
AMA	negative	
SMA	1:80	
LKM-1	negative	
Complement Component C3[mg/dL]	198.4	85-160
Complement Component C4[mg/dL]	25.3	12-36
IgG [mg/dL]	1053	716-1711
Adrenocorticotropic hormone [pg/mL]	28.07	7.2-63.6
Cortisol [μg/dL] 6 am	20.4	4.3-22.4
Cortisol [μg/dL] 7 pm	2.2	<16.66
FSH [mIU/mL ]	5.24	1-7.4
LH [mIU/mL ]	7.34	0.5-15
Estradiol [pg/mL]	46.75	25-345
Progesterone [ng/mL]	0.56	0.55-12.3
Testosterone [ng/dL]	40.78	28-1110
SHBG [nmol/L]	17.55	26.1-100
17-OH-Progesterone [ng/mL]	2.33	1-4.5
DHEA-S [μg/dL]	204.9	33.9-280

In abdomen ultrasound examination we found enlarged steatotic liver, splenomegaly and polycystic ovaries. The percutaneus liver biopsy revealed chronic diffuse steatohepatitis.

Although, elevated blood pressure was noted during routine measurement, there were no abnormalities in 24-hour blood pressure monitoring, echocardiography and electrocardiogram.

Radiological evaluation of the skeletal system showed no specific findings. No abnormalities were found during ophthalmological examination.

The clinical picture strongly suggested FPLD 2. We excluded acquired causes of lipodystrophy (HIV infection, deficiency of C4 and C4 complement components).

**Fig. 3:**
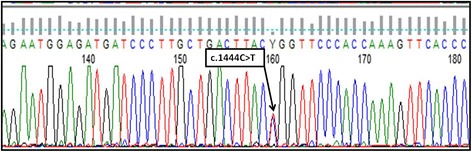
Result of DNA Sanger sequencing analysis of the *LMNA* gene: within ex. 8 the single nucleotide substitution C > T in one *LMNA* allele has been identified which is related to occurrence of p.Arg482Trp mutation in described patient. Sequencing result has been analyzed with usage of the Mutation Surveyor software

Genetic testing was performed to confirm FPLD 2 diagnosis. In order to perform the first step of genetic analysis 5 *LMNA* gene exons were selected (ex. 6,7,8,9,10). This selection was based on current database and literature findings showing that molecular defects of 3’ gene region are often related to the clinical outcome of FPLD 2. After prior amplification (PCR reaction) of selected regions, the direct DNA sequencing based on Sanger method was performed and the pathogenic mutation R482W (HGVS nomenclature: NM_170707.2: c.1444C > T; NP_733821.1: p.Arg482Trp) was identified in one allele of the *LMNA* gene (heterozygous form). R482W mutation has been registered in The Human Gene Mutation Database as a disease causing *LMNA* variant. Therefore, due to the disease autosomal dominant mode of inheritance, obtained genetic analysis confirmed the clinical diagnosis of FPLD 2 in our patient. Result of the DNA Sanger sequencing analysis of the *LMNA* gene in our patient is presented in Figure 3. Other members of our patient’s family did not consent for any clinical and genetic testing.

The girl was referred to the Department of Paediatric Metabolic Diseases, Children’s Health Institute in Warsaw for further care.

## Discussion

The estimated incidence of FPLD 2 is 1 case per 15 million persons [1]. However, the real prevalence may be 1 out of 200, 000 [6]. Discrepancies in morbidity data probably result from under-diagnosis of FPLD2 because of its heterogeneous phenotype mimicking metabolic syndrome, type 2 diabetes or Cushing syndrome [1]. To date, there have been described three cases of FPLD 2 in Poland [7–9].

In patients with FPLD 2, progressive loss of adipose tissue starts in puberty and concerns extremities and trunk making the muscles and veins of limbs more prominent. Significant sign of FPLD 2 is pseudohypertrophy of calves. Patients acquire athletic appearance [1, 10]. However, excessive accumulation of body fat in the face and neck may give Cushingoid appearance. Adipose tissue is also deposited in intra-abdominal region, and in women in vulva region [1]. Our patient’s phenotype is consistent with previous observations of FPLD 2 patients reported in the literature.

Skeletal abnormalities include short lower extremities with the height-to-legs-length ratio more than 2, greater bi-acromial than bi-trochanteric distance, broad hands with spindle-shaped fingers, and posture anomalies [1]. Retraction of Achilles tendons, deficiency of the pelvic and shoulder girdle, myalgia, muscular weakness or cramps may be also seen [1, 11, 12]. Our patient did not present these symptoms.

The most striking skin manifestation of FPLD 2 is acanthosis nigricans localized around the neck, in axillae and periumbilical area, which is a manifestation of insulin resistance. Other skin signs include seborrhoea, acne, leuko-melanoderma, subcutanues lipomas [1]. In our patient acanthosis nigricans and massive acne were the reason for medical consultation.

Metabolic derangements in FPLD 2 result from lipotoxicity of spared adipose tissue and include insulin resistance, diabetes and dyslipidemia, steatohepatitis, hyperandrogenism, and polycystic ovary syndrome [4]. In our patient, results of diagnostic tests revealed hyperinsulinaemia with insulin resistance, impaired glucose tolerance, steatohepatitis, hypertriglyceridemia and hypercholesterolemia. She had also significantly decreased adiponectin serum level. Serum leptin level was within laboratory ranges. However, serum leptin level in our patient was less than 10 μg/L, which is 69 % sensitive and 78 % specific for lipodystrophy [1].

In FPLD 2 cardiovascular complications are also presented i.e. cardiomyopathy, hypertension, early atherosclerosis and microangiopathy. Our patient had the incident of elevated blood pressure. However, other cardiovascular derangements may appear in the future [1, 12].

In women with FPLD 2 fertility and obstetrical complications are more common than in general population. Vantyghem et al. showed that in LMNA-mutated women the prevalence of polycystic ovary syndrome was 54 %, infertility 28 %, miscarriages 50 %, gestational diabetes 36 %, and eclampsia and foetal death 14 % [13]. Our patient exhibited a clinical phenotype of polycystic ovary syndrome.

The molecular background of lipodystrophy in our patient is the *LMNA* gene defect: R482W (p.Arg482Trp) mutation which has been described as a pathogenic variant responsible for FPLD 2. The first reported FPLD mutation of the *LMNA* gene was change at codon 482 in exone 8, which predicted the replacement of arginine by glutamine (Arg482Gln) [14]. Subsequently, Shackleton et al. identified five different missense mutations in *LMNA* gene i.e. Arg482Trp, Arg482Gln, Arg482Leu, Lys486Asn, Lys486Asn in ten kindred and three individuals with familial partial lipodystrophy [15].

More than ten different clinical syndromes have been attributed to *LMNA* mutations like FPLD 2, congenital muscular dystrophy, dilated cardiomyopathy type 1A [2] The wide phenotypic heterogeneity of diseases resulting from a mutation in a single gene may be explained by the variable roles of the nuclear lamina [2]. It has been observed that the majority of mutations in FPLD 2 affect the C-terminal domain of the lamin A/C protein, whereas alterations responsible for dilated cardiomyopathy and other diseases are usually clustered in the rod domain of the protein [16]. Mutations causing classical FLPD 2 usually affect “hot-spot” codon R482 which is probably responsible for decreased charge of specific surface on the C-terminal domain of lamin A/C. However, even in patients with the same *LMNA* genotype the clinical heterogeneity is significant [17].The CARE checklist is available as Additional file 1. 

## Conclusions

The diagnosis of FPLD 2 is based on the typical signs and symptoms, requiring thorough medical approach to a patient and targeted genetic analysis. Presented case shows the importance of precise and wide clinical description of patient’s outcome which indicates the optimal molecular diagnostic procedure, especially in such cases as *LMNA* gene which different mutations are responsible for multiple disorders. FPLD 2 should be considered in the differential diagnosis of diabetes, dyslipidemia, steatohepatitis, acanthosis nigricans and polycystic ovary syndrome.

## Consent

Written informed consent was obtained from the patient’s parent for publication of this Case Report and any accompanying images.

## Additional files

Additional file 1: CARE checklist. (DOC 1.51 MB)

## Abbreviations

HGVS: Human Genome Variation Society
FPLD 2: Familial partial lipodystrophy of the Dunnigan type
LMNA: lamin A/C gene
